# An Insight into Compositionally Complex Carbide Ceramic Coatings

**DOI:** 10.3390/ma18173953

**Published:** 2025-08-23

**Authors:** J. G. Lopes, J. P. Oliveira

**Affiliations:** CENIMAT/I3N, Department of Materials Science, NOVA School of Science and Technology, Universidade NOVA de Lisboa, 2829-516 Caparica, Portugal

**Keywords:** coatings, high-entropy carbides, medium-entropy carbides, compositionally complex ceramics, entropy-stabilized ceramics, carbides

## Abstract

Ceramic carbide coatings function as protecting components when subjected to extreme mechanical and/or high-temperature conditions. In this regard, the literature emphasizes that the compositionally complex design concept can be employed to improve the ceramic coating properties via compositional tuning, similarly to high-entropy alloys. At this moment, such studies are mainly based on the development of tribological coatings to obtain durable and low-friction surface barriers and to produce ablation-resistant barriers by forming stable oxide scales with self-healing mechanisms. Moreover, it can also be observed that the integration of computational design methods to predict and accelerate the discovery of optimized compositionally complex carbide ceramic coating systems is a viable possibility.

## 1. Introduction

### 1.1. The Role of Coatings in Surface Protection and Enhancement

Coatings are of extreme value for modern industry, as they can be used in a plethora of applications to enhance surface properties, improve esthetics, and grant protection against external elements. In other words, these are essential for protecting infrastructure, enhancing component performance, and promoting sustainability, provided that without them, modern industry and daily life would face higher costs, shorter material lifespans, and increased safety risks due to an increasing possibility for material failure and need for repairs, drastically impact long-term maintenance costs [1].

Drawing from the industrial point of view to a materials science perspective, coatings consist of a protective layer that is applied to a material surface, often referred to as substrate, in order to modify it. Of special interest, ceramic coatings are relevant for engineering applications, enveloping high melting points, high hardness, high compressive strength, and chemical inertness. Such properties are granted by strong ionic or covalent bonds, often both, that result in high bond dissociation energy and a stable crystal structure, which also account for ceramic low ductility and low tensile strength. More specifically, while ionic bonds are characterized by electron transfer occurring between metallic and non-metallic elements, covalent bonds correspond to when non-metallic atoms share electrons to form a compound. Such bonds directly relate to the properties exhibited by the coating, albeit not being the only factor that affects it [2].

Nevertheless, ceramic coatings are not a recent innovation, as they have been utilized for decades in industry. There are many types of ceramic materials that can be used as coatings, such as oxides (Al_2_O_3_ [3,4], ZrO_2_ [5,6]), carbides (SiC [7,8,9], WC [10,11]), nitrides (TiN [12,13]), and borides (TiB_2_ [14,15,16]) amidst many other options, each offering distinct advantages and trade-offs depending on the application [2]. Of specific interest to this review, carbide ceramics are known for their wear resistance through a combination of hardness and toughness, exceling in abrasive and high-load mechanical environments.

However, novelty on the topic has been mainly focused on manufacturing technologies within the scope of conventional ceramics, as highlighted in [17,18], showcasing their advantages and associated challenges. There is, however, an insurgence within the current literature that indicates that new coatings can be created based on the high-entropy concept (which has been relevant in the topic of materials science for the past two decades [19]). These may allow for the mitigation of challenges associated with carbide coatings, namely those related to their performance under high temperatures or durability due to crack propagation [20]. Therefore, these offer new opportunities for engineering applications and research efforts within the topic of carbide ceramic coatings.

### 1.2. A Brief Explanation of the Concept of High Entropy in Ceramics

The first studies regarding the concept of high entropy were published in 2004 by two independent research groups [21,22] regarding the possibility of producing novel and advanced metals, namely high-entropy alloys (HEAs). These were firstly presented to be composed of at least five principal components, where the term “high entropy” refers to the entropy of mixing, which helps stabilize a simple solid solution phase [23]. Such a requirement broadened with the increasing scientific appeal of the topic, where the idea of customizable alloy compositions for application-specific purposes, which eventually led to the establishment of three classes of metals, with these being low-entropy, medium-entropy, and high-entropy alloys, with the metals of the latter two classes fitting in a wider classification system, known as compositionally complex alloys (CCAs) or multi-principal element alloys (MPEAs). Nevertheless, since the topic of research is still relatively new, all these nomenclatures are widely accepted within their devoted research niches.

This leads us to the concept of compositionally complex ceramics (CCCs), whose definition is more complex due to the various physical attributes inherent to ceramics, which distinguish them from HEAs, which are characterized by purely metallic bonds.

According to Wright et al. [24], it is possible to define high-entropy ceramics (HECs) as compounds having at least five principal cations, with an entropy of mixing greater than 1.5 times the Boltzmann constant. Analogously to MEAs, there are the medium-entropy ceramics (MECs) which may overlap with the HEC class, depending on the number of cation sublattices of a structure. Both belong to the ionic bond type of ceramics. Other classes may also arise to include the predominant covalent bond type of ceramics, which is nevertheless still being included in the much wider CCC class. Moreover, a different definition by Rost et al. [25] mentions the entropy-stabilized oxide class, which implies the term entropy-stabilized ceramics and overlaps with all the aforementioned classes of ceramics. In other words, indicating that the entropy-stabilized phase is a result of high-entropy mixing of a compound on the Gibbs free-energy mixing overcomes its corresponding enthalpic contributions. Therefore, it is possible to scrutinize that the nomenclature given to these novel ceramics is still under thorough examination in research, provided that the boundaries between each terminology are not yet rigorously defined.

Nevertheless, while various types of ceramics can be identified and categorized into subgroups, this review specifically focuses on compositionally complex carbides.

## 2. The Current State of the Literature on Compositionally Complex Carbide Coatings

The existing literature reveals that research on compositionally complex carbides to date is primarily focused on their ablation resistance and tribological performance. As such, in this section, we aim to highlight the key studies and showcase the most significant recent developments related to each of these topics.

### 2.1. Ablation-Resistant Compositionally Complex Carbide Coatings

The most reported topic in the literature regarding compositionally complex carbide coatings refers to their ablation resistance properties, especially concerning the (HfTiZr)C system. Such a coating aims to protect against the loss of material from the surface, often caused by high-temperature environments, involving oxidation, evaporation, melting, and physical erosion of the coating material. As a matter of fact, although based on carbide systems, such coatings aim to produce a robust and self-healing oxide scale when exposed to high temperatures.

**Figure 1:**
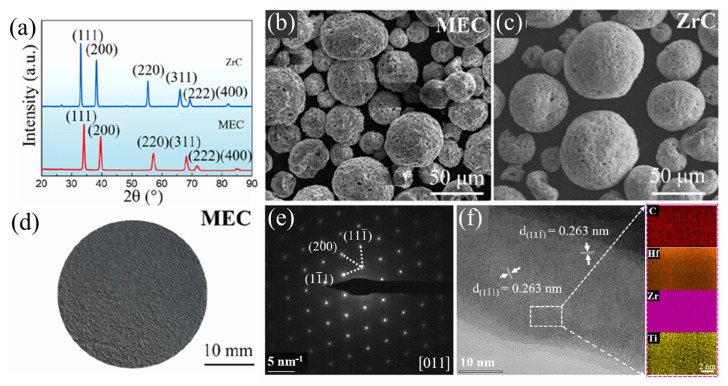
(**a**) X-ray diffraction patterns of MEC and ZrC powders; (**b**) (HfTiZr)C MEC powders; (**c**) ZrC powders; (**d**) macroscopic morphology of the (HfTiZr)C MEC; (**e**) SAED; and (**f**) high-resolution TEM micrograph alongside the corresponding element maps of the as-synthesized MEC. (Adapted from [26]).

A pioneering study on a particular topic of ablation-resistant compositionally complex carbide coatings was conducted by Li et al. [26]. There, a face-centered cubic (FCC) structured (Hf_1/3_Zr_1/3_Ti_1/3_)C coating was produced, which conveyed excellent high-temperature structural stability capable of enduring exposure to an oxyacetylene flame reaching temperatures higher than 2100 °C for over 210 s. Such results were compared to a ZrC coating, as observed in Figure 1. Moreover, transmission electron microscopy (TEM), as shown in Figure 1e,f, confirmed the FCC crystal structure through selected area electron diffraction (SAED) and revealed a consistent d-spacing, while element mapping demonstrating uniform composition at the atomic scale, verifying the formation of a single-phase solid solution MEC.

Interestingly, when exposed to the ablation tests, the advanced coating’s mass and linear ablation rates showcased a decrease from 1.54 mg/s to 0.15 mg/s and 2.54 μm/s to 0.19 μm/s with increasing time (ranging from 30 and 210 s). Moreover, the resulting oxide scale exhibited an amorphous Zr–Hf–Ti–C–O oxycarbide pinned by (Hf, Zr, Ti)O_2_ nanoparticles, which improved the long-term ablation resistance of the coating and hinted at the possibility for new-generation structural materials in ultra-high-temperature environments.

Moreover, subsequent studies delve deeper into the (HfTiZr)C system. Such is the case of Li et al. [27], where the slightly compositionally different (Hf_0.25_Zr_0.25_Ti0_.5_)C was tested as a coating on a C/C composite substrate with a SiC interlayer. Ablation resistance, microstructure, and defect initiation were investigated and coupled with theoretical simulation analysis, revealing the formation of Ti-doped (Hf, Zr)O_2_ and (Hf, Zr)TiO_4_ on the surface after ablation. The combination of the properties exhibited by these compounds granted protection of the substrate during 120 s of ablation (above 2100 °C), while stress concentration resulted in the formation of damaged areas at their interfaces. In this case, with increasing ablation time, ranging from 60 s to 120 s, an increase in both the mass ablation and the linear ablation rates was observed, ranging from −1.05 mg/s to −0.82 mg/s and −0.67 μm/s to 0.67 μm/s, respectively.

Interestingly, Li et al. [28] reported the martensitic transformation potential of Hf/Zr-rich oxides and the sealing role of Ti-rich oxides resulting from exposure to (Hf_1/2_Zr_1/4_Ti_1/4_)C and (Hf_1/4_Zr_1/2_Ti_1/4_)C coatings in severe thermal environments. This allowed the authors to further elucidate the twin toughening-driven martensitic transformation mechanisms and failure behaviors that grant such types of coatings excellent performance. The mechanisms were prompted via the “slip band-twin transfer” and “stacking fault-twin transfer” within Ti-doped (Hf_2/3_Zr_1/3_)O_2_ and Ti-doped (Hf_1/3_Zr_2/3_)O_2_, thus improving the thermal shock resistance of the coatings. Such in-depth analysis is consistent with previous studies on the matter and managed to grant protection up to temperatures of 2180 °C in multiple 120 s ablation cycles.

Furthermore, with a focus on optimizing the ablation resistance of the (HfTiZr)C system while considering the amount of reaction products during ablation, Song et al. [29] explored different molar ratios of Hf, Ti, and Zr elements. Once again, the results were compliant with those exhibited previously, where the influence of compositional ratio control on the ablation behavior and mechanisms of carbide ceramics were analyzed, proving the synergistic structural and self-healing effect of the resulting multi-component complex oxide layers and providing the foundation for high-temperature thermal protection.

With these advances, combinations involving the compositionally complex carbide (HfTiZr)C and ultra-high-temperature ceramic-modified C/C composites have also been studied in the literature [30,31,32,33].

Nevertheless, other elements have also been considered within the scope of developing compositionally complex carbide ceramic coatings. Analogously, a carbide system coating, composed of (Hf_1/3_Zr_1/3_Ta_1/3_)C, was investigated by Zhang et al. [34], exhibiting an ablation resistance above 2000 °C. Such a coating was deposited via supersonic atmospheric plasma spraying in a SiC-coated C/C composite, resulting in a dense FCC ceramic. With consequent ablation at 2100 °C for 120 s, the coating exhibited a low linear ablation rate of −0.61 μm/s, which resulted in the formation of a compact oxide scale consisting of (Hf, Zr)O_2_, which functioned as a skeleton and (Hf, Zr)_6_Ta_2_O_17_, which melted to fill its cracks. Nevertheless, it was hinted that excessive oxide melt resulted in the instability of the oxide scale, thus requiring subsequent compositional optimization to improve the coating’s resistance to ablation.

Likewise, Zhang et al. [35] reported the ablation resistance of three refractory carbide ceramic coatings, including (HfTi)C, (HfTiZr)C, and (HfTiZr)C–TaC coatings prepared by the molten salt method. The EDS data revealed a composition of Zr (8.2%) and Hf (47.7%) for the (HfTi)C coating; Zr (8.5%), Ti (7.7%) and Hf (6.3%) for the (HfTiZr)C coating; and Ta (10.5%), Ti (9.3%), Zr (6.5%) and Hf (5.8%) for the (HfTiZr)C–TaC coating. After exposure to an oxy-acetylene torch, it was noted that the coatings had good ablation resistance, where the highest ablation temperature of 1790 °C and 1830 °C with corresponding mass ablation rates of 0.61 mg/s and 0.51 mg/s were attained for the (HfTiZr)C and (HfTiZr)C–TaC coatings, respectively. Therefore, it was possible to achieve due to the formation of ZrO_2_ and HfO_2_ with high melting points, which provided structure, combined with TiHfO_4_ and Ta_2_O_5_ with low melting points, which played a self-healing role, thus resulting in the formation of composite oxide ceramic layer capable of enhancing the coating’s resistance to ablation.

**Figure 2:**
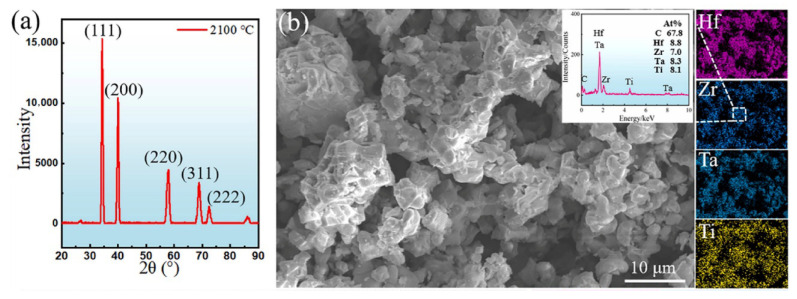
The structural and compositional results of synthesized (Hf_1/4_Zr_1/4_Ta_1/4_Ti_1/4_)C HEC powders at 2100 °C: (**a**) X-ray diffraction pattern; (**b**) scanning electron microscopy images and energy-dispersive spectroscopy analysis results. (Adapted from [36]).

Similarly, Li et al. [36] evaluated the long-term ablation resistance of an FCC (Hf_1/4_Zr_1/4_Ta_1/4_Ti_1/4_)C as an advanced anti-ablation coating, with a focus on analyzing the oxidation reaction of action to protect the substrate, which is presented in Figure 2. It was found that the oxygen atoms preferentially adsorbed at sites where Hf and Zr aggregated, forming a Hf-Zr-rich monoclinic oxide, (Hf, Zr)O_2_. The subsequent diffusion of TiO_2_ and Ta_2_O_5_ into these regions led to the formation of needle-like (Hf, Zr)_6_Ta_2_O_17_ and rounded (Hf, Zr)TiO_4_ particles. The interaction between these two phases promoted the development of a nanotwin structure, which significantly enhanced the toughness of the oxide film. Although an excess of Ta_2_O_5_ disrupted the overall stability of the oxide, the skeleton, composed of Ta/Ti-doped (Hf, Zr)O_2_, (Hf, Zr)_6_Ta_2_O_17_/(Hf, Zr)TiO_4_, effectively protected the substrate during 180 s of ablation.

**Figure 3:**
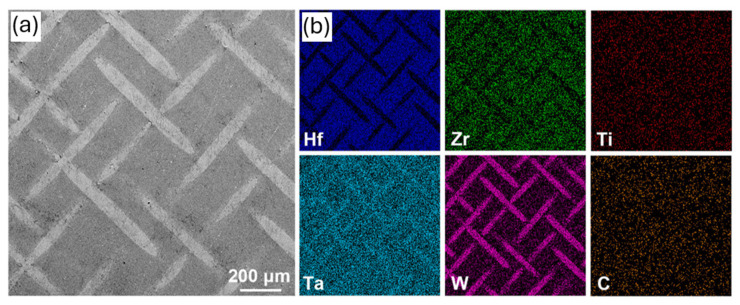
(**a**) Scanning electron microscopy highlighting the W mesh embedded in the HfZrTiTa carbide ceramic coating and (**b**) corresponding energy-dispersive spectroscopy maps (adapted from [37]).

Moreover, Li et al. [37] designed the (Hf_0.5_Zr_0.3_Ti_0.1_Ta_0.1_)C-W with an integrated refractory tungsten mesh in order to achieve excellent ablation resistance in extreme environments, which can be observed in Figure 3. Remarkably, the coating remained intact when subjected to ablation at 2500 °C for 600 s, exhibiting a linear ablation rate of −1.75 μm·s^−1^ and a mass ablation rate of −0.149 mg·s^−1^·cm^−2^. Such performance is characteristic of the formation of (Hf, Zr, Ti)O_2_, which grants it its structure, while Ti-rich oxides, appearing as liquid phases, tend to fill the skeleton pores and form a dense oxide scale to prevent oxygen diffusion. Additionally, the Ta-rich oxides play a role in preventing crack growth and improving thermal shock resistance due to their lamellar structure. Overall, the coating achieved a high thermal conductivity of 25.72 W·m^−1^·K^−1^, resulting in a 16.7% enhancement to that of the pure (Hf_0.5_Zr_0.3_Ti_0.1_Ta_0.1_)C ceramic system. Interestingly, tests also indicated that at higher ablation temperatures up to 2700 °C for 600 s, the coating still remains intact. Synergistically, the W mesh may also impede crack propagation while improving the thermal protection performance of the coating.

Focusing on the (HfZrTaNb)C system, Li et al. [38] optimized the (Hf_0.25_Zr_0.25_Ta_0.25_Nb_0.25_)C coating composition, which exhibited poor ablation resistance at 2000 °C due to a nearly equal ratio of high- and low-melting-point phases. The optimal coating corresponded to (Hf_0.45_Zr_0.45_Nb_0.1_)C, achieving a linear ablation rate of −0.01 μm/s for over 300 s. This was possible by reducing the Ta or Nb content, thus significantly improving the coating performance, as more stable (Hf, Zr)O_2_ skeleton phases were obtained within the oxide scale.

Using a different approach, Guo et al. [39] proposed using the concept of inverse design, thus starting by pre-selecting an oxide layer with desired properties, followed by assuring its formation upon ablation of the (Hf_0.36_Zr_0.24_Ti_0.1_Sc_0.1_Y_0.1_La_0.1_)C coating. The goal was to enable the formation of an oxide protection layer with pre-designed stability and mechanical strength for enhancing ablation resistance. This research study demonstrated the effectiveness of the inverse theoretical design, providing a novel optimization approach for the ablation protection of high-entropy carbide coatings. Other approaches envisage utilizing first-principles calculations for simulating and understanding the mechanisms underlying surface oxidation and the microstructural evolution of oxide layers at high temperatures. Such was the research conducted by Tang et al. [40] in an FCC (TiZrNbMoAl)C coating, where simulations and experimental results hinted at the formation of a multilayer oxide structure, where O atoms tend to occupy the hollow FCC sites with higher concentrations of strong carbide-forming elements (Ti, Zr) to form stable oxides, followed by weak carbide-forming elements (Al, Mo). Thus, this indicates that Ti concentration must be controlled in order to improve the oxidation resistance of the coating.

### 2.2. Tribology-Focused Coatings Composed of Compositionally Complex Carbides

With the rapid development of industry, which consistently integrates more complexity into its products, it is important to consider the reduction in friction and wear, which has a direct impact on improving energy consumption and component lifespan through the selection and optimization of tribological systems [41].

Regarding this matter, research has been conducted to achieve stable and advanced compositionally complex carbide coatings with the continuous enhancement of such engineering purposes in visage.

For instance, Braic et al. [42] presented a comprehensive assessment on the tribological properties of the (TiZrNbHfTa)N and (TiZrNbHfTa)C coatings with TiN, TiC, and metallic TiZrNbHfTa films as reference. It was observed that both coatings exhibiting an FCC solid solution with a strong (111)-oriented texture exhibited higher hardness values than their reference counterparts. Further analysis via X-ray photoelectron spectroscopy (XPS) revealed pure and intermetallic chemical states between the metallic elements, confirmed by distinct binding energy peaks, with uniform metal distribution and consistent non-metal/metal ratios. Complementary Raman and XRD data indicated the formation of a nanocomposite structure composed of nanocrystalline carbides and amorphous carbon phases.

Overall, the (TiZrNbHfTa)C coating showcased the best tribological performance, with a low friction coefficient (≈0.15) and a wear rate of 0.8 × 10^−6^ mm^3^/Nm, making it a promising option for wear-resistant applications.

Moreover, Sun et al. [43] reported the single-phase FCC (HfMoNbTaTi)C coating. Comparing its performance to NbC, the self-lubricating mechanism originates from the formation of a carbon-rich film with self-lubricity between the intervenient wear surfaces, which is generated through the diffusion and enrichment of carbon produced during friction. The experiments included different tribo-pairs, comprising Al_2_O_3_ and Si_3_N_4_ balls, and they were performed under loads of 10 N, 20 N, 30 N, and 50 N while exposed to different levels of frequency (1.5 Hz, 3 Hz, 5 Hz, and 7 Hz). The results indicate that the most influential parameters on the friction coefficient are the applied loads and the tribo-pairs, while the sliding frequency has less influence. The minimal friction coefficient obtained was 0.3, (tested with the Si_3_N_4_ balls under 30 N and 5 Hz). Moreover, it was also concluded that the wear rate and friction coefficient tend to decrease by increasing the applied load up to 30 N, whereas the wear rate was calculated to be within the range of 10^–7^~10^–6^ mm^3^/Nm.

**Figure 4:**
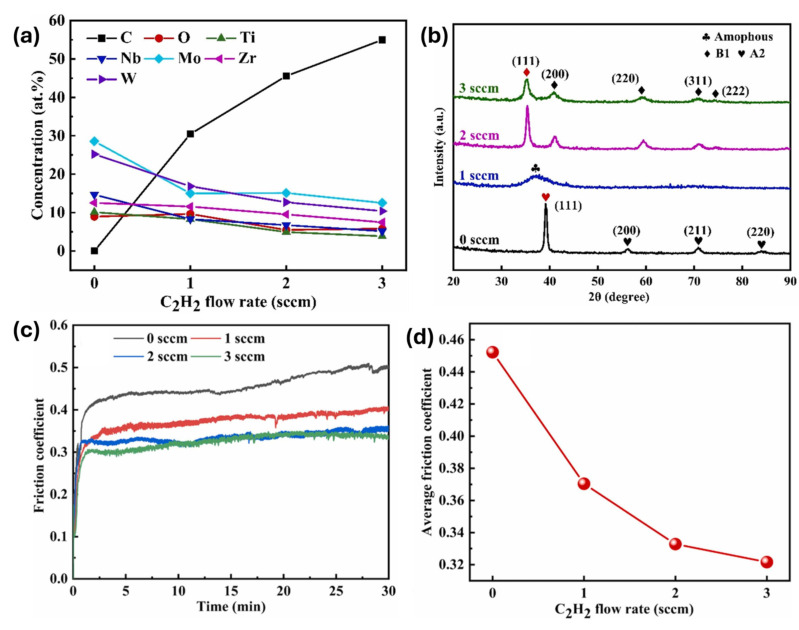
(**a**) Elemental composition of (TiNbMoZrW)C coatings probed by scanning electron microscopy as a function of C2H2 flow rates; (**b**) X-ray diffraction patterns of the (TiNbMoZrW)C coatings deposited at different C2H2 flow rates; (**c**) friction coefficient curves and (**d**) average friction coefficient (TiNbMoZrW)C coatings (adapted from [44]).

Wang et al. [44] examined the resulting tribological properties with an emphasis on process parameter optimization through the impact of the C_2_H_2_ flow rate on (TiNbMoZrW)C coatings during magnetron sputtering. From their experiments, it was concluded that with the increasing flow rate (ranging from 0 sccm to 3 sccm), the deposition rate of the coating increased, as well as the carbon content within the coating, which reached a maximum of ≈55 at. % (refer to Figure 4a). Furthermore, as observed in Figure 4b, the transition in crystal structure from FCC to an amorphous structure to a body-centered cubic (BCC) structure was observed to occur in relation to the C_2_H_2_ flow rate. Moreover, further analysis via XPS revealed that coatings deposited without C_2_H_2_ contained metallic and metal–oxide bonds, whereas at a C_2_H_2_ flow rate of 1 sccm, coatings exhibit a mix of metallic, metal–oxide, and metal–carbide bonds. At higher C_2_H_2_ flow rates (above 1 sccm), only metal–oxide and metal–carbide bonds are present, with metallic bonds completely disappearing. Interestingly, observing Figure 4c, it can be seen that all the coatings exhibited low friction coefficients and good wear resistance, with the one deposited at 2 sccm possessing the best tribological properties, showcasing an average friction coefficient of 0.33 and a minimum wear rate of 3.9 × 10^−7^ mm^3^/Nm. The same trend was observed in terms of hardness, elastic modulus, and indentation toughness.

Using a different approach, Shi et al. [45] used machine learning to design and choose the optimal elemental ratio of the (NbTaMoWAl)C system (with special focus on Al) to predict its mechanical properties, thus intending to optimize coating performance. Two coatings were fabricated via pulsed DC magnetron sputtering, the (NbTaMoW)C and the (NbTaMoWAl)C, with an Al content of ≈ 9.5 at. %. From the results, the (NbTaMoWAl)C coating showcase both a lower friction coefficient and wear rate (approximately 2.65 × 10^−6^ mm^3^/Nm) when compared to the (NbTaMoW)C case. Such was a product of the 18% increase in hardness exhibited by the (NbTaMoWAl)C coating, which arises as a consequence of lattice distortion and solid solution strengthening factors (inherent to compositionally complex materials), thus improving the coatings’ resistance to crack damage and plastic deformation. Such research highlights the potential of compositional tuning via the high-entropy concept for the optimization of coating properties.

**Figure 5:**
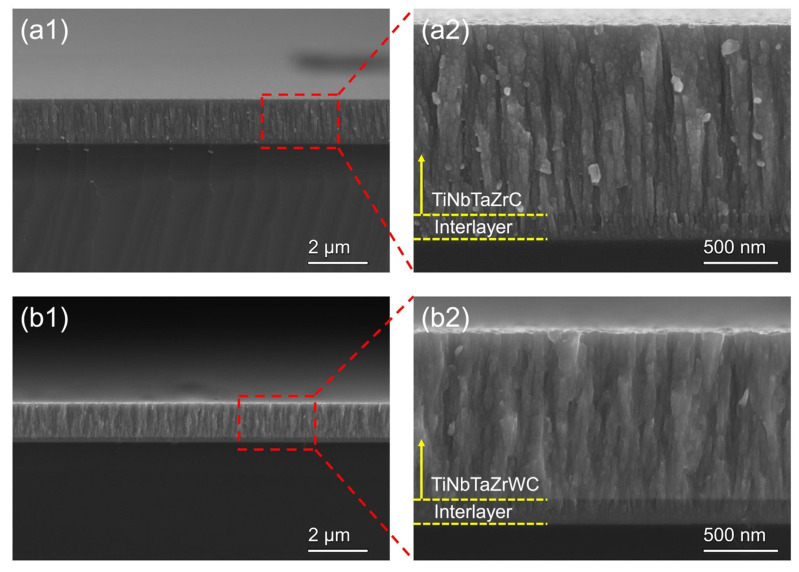
Scanning electron microscopy of the cross-section of the (**a1**) (TiNbTaZr)C coating with a corresponding (**a2**) magnified view and (**b1**) (TiNbTaZrW)C coating and a corresponding (**b2**) detailed view (adapted from [46]).

Similarly but on a slightly different topic, the tribo-corrosion properties of (TiNbTaZrW)C as coatings for the protection of medical tool surfaces were investigated by Gao et al. [46], focusing on machine learning to optimize the composition ratio and improve wear resistance. For their study, a compositional ratio of 10:10:7.5:7.5:15:50 at. % was attained and deposited using pulsed DC magnetron sputtering technology. The tribological properties were then tested in simulated body fluid and compared to the properties exhibited by a (TiNbTaZr)C coating. The coating exhibited excellent wear resistance and lubricating properties as a result of the microstructure observable in Figure 5, where the (TiNbTaZrW)C exhibits a denser structure than the (TiNbTaZr)C coating. Excellent corrosion resistance was also observed in the coating granted by the formation of a passive film resulting in a corrosion current density of ≈6.87 × 10^−8^ A/cm^2^. Nevertheless, due to the excellent resistance to crack damage and plastic deformation, the (TiNbTaZrW)C coating exhibited a wear rate of 2.34 × 10 mm^3^/Nm, thus exhibiting excellent wear resistance and biocompatibility.

From a high-temperature tribology perspective, Li et al. [47] synthesized, using spark plasma sintering, a (TiZrVNb)CF compositionally complex carbide ceramic with a single-phase BCC structure. The coating was compared to conventional ZrC ceramics over a broad temperature range (25 °C to 900 °C), showcasing significantly improved mechanical and tribological properties, which was attributed to the solution strengthening and lattice distortion effects. Overall, at room temperature, a 0.2 friction coefficient and excellent wear resistance (wear rate = 9.55 × 10^−7^ mm^3^/N·m) were attained, granted by the high load-bearing capacity and the formation of a protective glaze layer on the counterpart surface, playing the role of a friction and wear barrier. At elevated temperatures, the advanced coating maintained superior wear performance due to its high thermal stability and oxidation resistance, which is possible due to the formation of a multi-layered complex oxide film, acting as an effective barrier against adhesive wear and brittle fracture. Consequent XPS analysis also showed that the carbide coating undergoes partial oxidation at 25 °C, forming oxides such as TiO_2_, ZrO_2_, and Nb_2_O_5_. At 300 °C, the same types of oxides tend to form, although at the carbide matrix, it disappears. As the temperature rises to 600 °C and 900 °C, only fully oxidized compounds are present on the surface, which is related to the enhancement of the wear resistance of the coating.

From a more mechanical property-focused perspective, Sun et al. [48] focused on how different elements affect the formation of high-entropy phases, regarding (TaZrTiNbV)C and (TaMoTiNbV)C as coatings produced via plasma spraying. Due to differences in thermal conductivity, (TaZrTiNbV)C exhibited a higher degree of supercooling, resulting in finer grains, while (TaMoTiNbV)C resulted in more time for grain growth to occur. Eventually, it was shown that (TaMoTiNbV)C exhibited a higher hardness due to complete solid solution and lattice distortion, whereas (TaZrTiNbV)C featured more carbide particles, which enhanced its toughness.

## 3. Discussion and Future Perspective

As highlighted, coatings play an important role in enhancing the performance and longevity of components when these are exposed to harsh service conditions. This ensures long-term stability, adaptability and cost-effectiveness, thus driving innovation in protective coatings.

**Figure 6:**
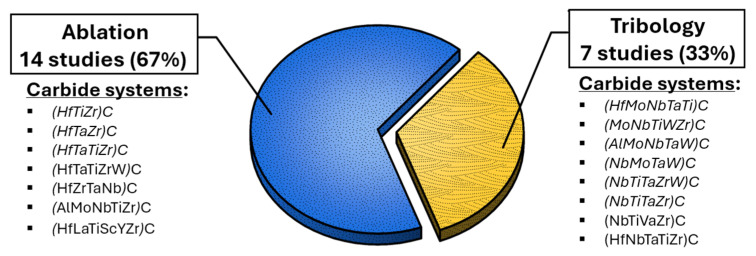
Percentage distribution of the number of papers considered in this study.

As a result, compositionally complex carbide coatings have gained particular relevance in the field, with current research trends focusing especially on tribological and ablation-related applications, as can be seen in Figure 6, showcasing the distribution of publications analyzed in this study. Two thirds of the studies are dedicated to ablation-resistant coatings.

Regarding ablation-resistant coatings, which are essential in applications where materials are exposed to extreme temperatures, the literature shows that most research is being conducted to couple the formation of a stable oxide scale, with the formation of self-healing oxides, therefore reducing material loss during ablation and improving coatings’ protective and repair features. This is made possible by the composition tuning of the compositionally complex design concept, which allows us to combine different properties in a single-carbide system.

In the field of tribology, compositionally complex carbide coatings are especially valuable, as they can form strong and stable barriers that can endure extreme tribological conditions by reducing friction and wear between contacting surfaces. The literature indicates that, in this context, a more diverse set of systems has been studied, thus highlighting the versatility and potential for carbide coatings. Moreover, it was observed that their performance is directly affected by their composition and microstructure, which can be engineered on a nano- and microscale to enhance performance.

Moreover, compositional optimization was observed to influence their ablative and tribological properties by dictating the phase structure, composition, and bonding structures within the material. Consequently, precise control of elemental ratios is essential to optimize the balance between mechanical strength and resistance to high-temperature conditions in these advanced carbide materials.

Looking ahead, the integration of computational design and machine learning techniques holds promise for accelerating the discovery of novel carbide coating systems. Nevertheless, for the next generation of high-performing compositionally complex carbide coatings, it is necessary to further research their long-term performance and degradation mechanisms under extreme operating conditions.

## 4. Conclusions

In conclusion, compositionally complex carbide coatings have emerged as a promising solution to enhance the durability and functionality of components exposed to extreme environments, especially in tribological and high-temperature ablation environments.

As such, the adaptability of these coatings, enabled by compositional tuning, reflects the potential to tailor coating performance to specific applications, ensuring both resilience and efficiency. Moreover, with sustained research, compositionally complex carbide coatings have the potential to be the next generation of protective coatings.

## Data Availability

No new data were created or analyzed in this study. Data sharing is not applicable to this article.
